# Trend, application, and reporting of Mini-health technology assessment: an evidence mapping

**DOI:** 10.1017/S0266462325100585

**Published:** 2025-12-04

**Authors:** Ziyi Wang, Yafang Li, Meng Xu, Wendi Liu, Yilong Yan, Yongsheng Wang, Yanan Wu, Meixuan Li, Xiuxia Li, Xiaomei Zhang

**Affiliations:** 1 Lanzhou University, China; 2 Air Force Medical University, China; 3 Gansu Provincial Hospital, China

**Keywords:** Mini-Health Technology Assessment, Technology Assessment Biomedical, Evidence Mapping, Reporting Guideline, Review

## Abstract

**Objective:**

This study aims to systematically identify and summarize the key characteristics of Mini-Health Technology Assessment (Mini-HTA) and assess the completeness of its basic reporting information, providing a theoretical foundation for developing future reporting guidelines..

**Methods:**

A comprehensive search for Mini-HTAs was performed using CNKI, Wanfang Data, VIP, CBM, PubMed, Embase, Web of Science, HTA database, and major HTA-related websites from inception until February 2024. The completeness of basic information reporting in Mini-HTAs was assessed using the INAHTA checklist. The key characteristics of the included Mini-HTAs were summarized descriptively. Microsoft Excel 2019 was used to analyze and visually present the data.

**Results:**

A total of 21 Mini-HTA reports were included, with the highest number published in 2021 (5 reports, 23.8 percent). China contributed the most reports (18 reports, 85.7 percent). The most common assessment purpose was technology comparison (15 reports, 71.4 percent), with general hospitals being the predominant assessment setting (17 reports, 80.9 percent), and drugs being the most frequently assessed technology type (14 reports, 66.7 percent). The INAHTA checklist evaluation identified notable deficiencies in reporting key methodological aspects, including participant roles, conflict of interest statements, data sources, literature search strategies, and methods for data assessment and analysis.

**Conclusion:**

Mini-HTAs have significantly increased in China since 2020, mainly in technology comparison, drug evaluation, and general hospitals. However, gaps remain in reporting key aspects, such as participant roles, conflict of interest, and data sources. Future efforts should focus on refining reporting guidelines to improve consistency and address these reporting deficiencies in Mini-HTA.

## Introduction

The concept of Mini-Health Technology Assessment (Mini-HTA) originated from Copenhagen Hospital Corporation/Copenhagen University Hospital in Denmark, where it was first utilized to provide scientific evidence for decision-makers in approving new health technologies at clinical, organizational, and political levels (1). In 2005, the Danish Centre for Health Technology Assessment (DACEHTA) integrated experiences from the application of Mini-HTA in various contexts to develop a standardized tool for local and regional decision-making, establishing a systematic definition of Mini-HTA (1). Mini-HTA is a streamlined method based on evidence-based medicine and HTA principles, systematically assessing health technologies in specific settings to support decision-making on their adoption (2). While full HTA primarily addresses health issues of national priority, focusing on disease burden and policy considerations, Mini-HTA is designed to be more adaptable, particularly for settings like hospitals that require rapid decision-making and have more limited resources (3). Unlike full HTA, which often requires extensive time and high-quality evidence, Mini-HTA is refined in terms of assessment time, scope, and content, making it more suitable for resource-constrained settings (4). Although Mini-HTA can be implemented in hospitals, it is not limited to them. The original Mini-HTA table focused on assessing four dimensions: technology, patients, organization, and economics, and was designed to be applied to various specific settings (5). Recently, researchers have increasingly explored methodological innovations in Mini-HTA to enhance decision-making quality in specific settings (6, 7). For instance, Martelli et al. developed a novel decision-support tool that integrates Multi-Criteria Decision Analysis (MCDA) with Mini-HTA, demonstrating its effectiveness in fostering more structured and transparent HTA processes in specific settings (6).

As Mini-HTA continues to evolve, it has been increasingly adopted by healthcare institutions and policymakers, emerging as a valuable tool for health decision-making across various levels (7). However, poor-quality Mini-HTAs undermine their potential value in clinical and policy applications (8). Therefore, an increasing number of researchers use the HTA table developed by the International Network of Agencies for Health Technology Assessment (INAHTA) to assess quality of existing HTA reports, as this table is applicable to different types of HTAs. For example, Kidholm et al. used the INAHTA checklist to assess the quality of 52 Mini-HTAs conducted in Danish hospitals and found that information on the selection and interpretation of clinical literature and other data was often missing (8).Furthermore, Mini-HTA often lack clarity regarding reporting completeness, assessment criteria, and diseases distributions, while duplication of Mini-HTAs remains common (9-11).

## Methods

### Literature search

To address these challenges, evidence mapping is a novel synthesis method that systematically collects and analyzes evidence on a specific topic, evaluates and integrates findings, and presents them concisely to highlight the research status, challenges, future research directions, and evidence gaps (12). Therefore, we used this approach to conduct a comprehensive review of Mini-HTAs, aiming to evaluate their conduct a review of the current state-of-the-art of the structure and contents of Mini-HTA reports and provide valuable insights for their future application.

China National Knowledge Infrastructure (CNKI), Wanfang Data, VIP, Chinese Biology Medicine disc (CBM), PubMed, Embase, Cochrane Library, and the HTA database, along with major HTA-related websites (see in Supplementary Materials) were systematically searched by two independent reviewers (ZYW and YFL) from inception to 26^th^ February 2024. The Medical Subject Headings (MeSH) terms combined with keywords were used to establish the search strategy, and the main search words were “Biomedical Technology Assessment”, “Biomedical Technology Assessments”, “Health Technology Assessment”, “Health Technology Assessments”, “Mini”, etc. Futhermore, the reference lists of selected articles were manually searched to identify additional relevant publication publications. The full search strategy and specific web address for each database in show in Supplementary Materials.

### Eligibility criteria

Studies included in this review must meet at least one of the following criteria: (1) the study type is explicitly identified as Mini-HTA, or (2) the assessment criteria are clearly reported using the Mini-HTA table developed by DACEHTA, or an assessment form based on this table. Duplicate publications, conference proceedings, research protocols, articles not written in Chinese or English and those publications whose full texts could not be accessed were excluded.

### Literature selection and data extract

Duplicate records were removed using EndNote X20 before the formal screening. Two independent researchers (ZYW and YFL) then selected the Mini-HTA through a two-step process. First, the researchers screened the title and abstract of identified records to exclude irrelevant records. Second, the researchers obtained the full texts of the remaining records and identified eligible Mini-HTA. Any disagreement about inclusion was resolved by consensus or consultation with a third reviewer (XXL).

Two independent researchers (ZYW and YFL) abstracted the data using a predesigned data extraction table. The extracted information from Mini-HTA was included: (1) first author name, (2) the year of publication, (3) country of first author, (4) presence of HTA professionals (refer to individuals engaged in HTA or related fields, such as evidence-based medicine or health economics, based on the authors’ affiliations and departmental information), (5) number of authors, (6) purposes of assessment (classified into introduction of new technologies, comparison of technologies, application of existing technologies in new fields, decommissioning of technologies), (7) type of disease [classified according to International Classification of Diseases 11^th^ Revision (ICD-11) (13)], (8) assessment setting (classified into general hospital, specialized hospital, and primary healthcare institution), (9) assessment criteria (e.g., Mini-HTA table developed by DACEHTA), (10) type of technology (classified into drug, surgery, medical equipment, and others), (11) other information (e.g., the classification of journal, funding source). Disagreements were resolved by discussion or consultation with a third reviewer (XXL).

### Reporting of mini-HTA

The completeness of basic information reporting in Mini-HTAs was independently assessed by the two researchers (ZYW and YFL), and disagreements were resolved by a third reviewer (XXL). INAHTA checklist (2007) was used to assess the completeness of reporting basic information of the Mini-HTAs (14).This checklist consists of 14 items, and “Yes”, “Partial Yes”, or “No” options were used to assess the compliance of the mini-HTAs (14).

The checklist includes five questions about preliminary information (contact details, authors, conflict of interest, external review, summary), four questions about why the assessment has been undertaken (policy question, research question, scope, health technology), two questions about how the assessment has been undertaken (source of information and literature search strategies, assessment and interpretation of selected data), three questions about what then?-Implications of the assessment results and conclusions (discussion, conclusion, suggestion) (14).

In this study, items with a percentage of “Yes” lower than 50 percent were considered to indicate highly flawed areas in the included Mini-HTAs, highlighting areas that need substantial improvement in the future (15).

### Data synthesis and analysis

The basic characteristics and quality assessment of the included Mini-HTAs were summarized descriptively. We utilized Excel 2019 to create a bubble chart. The bubble chart was employed to visualize the distribution of results, primarily from four dimensions: report items, reporting extent, number of authors involved, and assessment criteria. Each bubble represents a Mini-HTA, with different colors indicating different assessment criteria. The size of the bubble reflects the number of authors contributing to each Mini-HTA. The X-axis represents the reporting extent of each item of the INAHTA checklist, while the Y-axis displays the different items of the INAHTA checklist.

## Results

### Literature screening process and results

**Figure 1.**
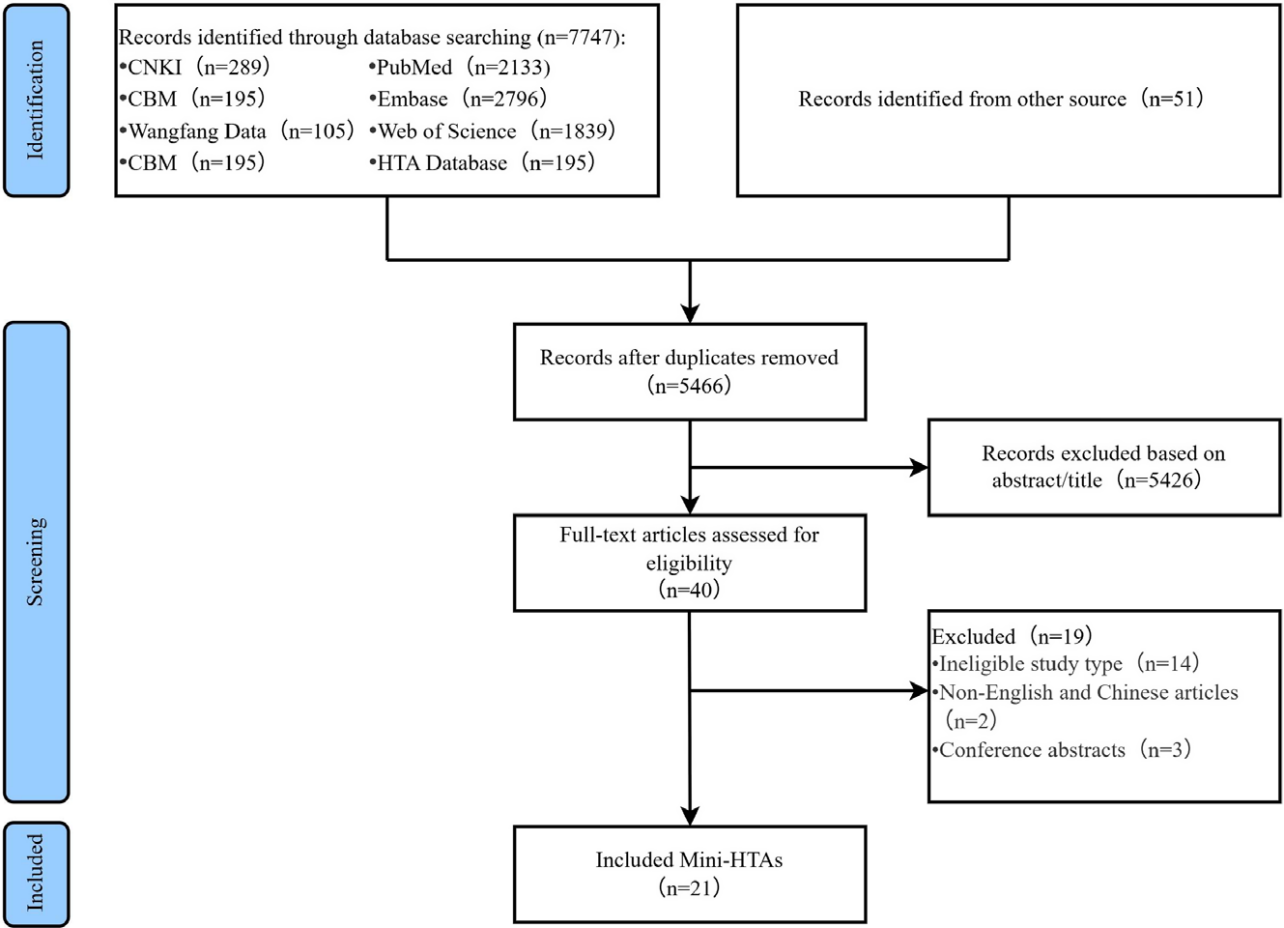
Flowchart of the systematic search and selection process. *Note:* Item 1, Are there appropriate contact details for provision of further information? Item 2, Are those who prepared the HTA report identified as authors or in other ways? Item 3, Is there a statement regarding conflict of interest? Item 4, Is there a statement on whether the report has been externally reviewed? Item 5, Is there a short summary that can be understood by the non-technical reader? Item 6, Is reference made to the policy question that is addressed? Item 7, Is reference made to the research question(s) that is/are addressed? Item 8, Is the scope of the assessment specified? Item 9, Is there a description of the health technology that has been assessed? Item 10, What sources of information have been used? Item 11, Is there information on the basis for the assessment and interpretation of selected data and information? Item 12, Are the findings of the assessment discussed? Item 13, Are the conclusions from the assessment clearly stated? Item 14, Are there suggestions for further action?

A flow diagram of the literature selection process is presented in Figure 1. A total of 7798 relevant records were initially identified. A total of 2332 of 7608 records were excluded because of duplication, and 5426 records were excluded based on their titles and abstracts. A total of 19 of the remaining 40 records were further excluded after reading the full texts. Finally, only 21 Mini-HTAs (4, 6, 9-11, 16-31) were included in this study.

### Characteristics of included mini-HTA

Among the 21 Mini-HTAs, the highest number was published in 2021 (n=5, 23.8 percent) (Table 1). The majority of first authors were from China (n=18, 85.7 percent), followed by Canada, South Africa, and France, each contributing one study (4.8 percent). Only 33.3 percent of Mini-HTAs (n=7) involved HTA professionals. In terms of assessment purposes, 15 Mini-HTAs (71.4 percent) focused on comparison of technology that a comparison of the two technologies that are already available in the hospital, while the remaining 6 (28.6 percent) were intended for the introduction of new technology, which the content is also compared with standard care. According to the ICD-11, the included Mini-HTAs covered seven disease categories, with endocrine, nutritional, and metabolic diseases being the most common (n=3, 14.3 percent), followed by diseases of circulatory system (n=2, 9.5 percent) and neoplasms (2 reports, 6.1 percent). Regarding assessment settings, 17 Mini-HTAs (81.0 percent) were conducted in general hospitals, while 4 (19.1 percent) were in specialized hospitals In terms of technology type, drugs were the most frequently assessed (n=14, 66.7 percent), followed by medical equipments (n=7, 33.3 percent). The 20 Mini-HTAs were published in 17 different journals, with only one sourced from an HTA database. The *Chinese Medication Journal*, *Chinese Journal of Pharmacoepidemiology*, and *China Pharmacy* had the highest number of publications, each contributing 2 Mini-HTAs (9.5 percent). 57.1 percent of Mini-HTAs (n=12) were published in general journals. In terms of funding, 11 Mini-HTAs (52.4 percent) did not mention funding information, while 10 Mini-HTAs (47.6 percent) reported receiving nonprofit funding.

**Table 1.** Summary of the characteristics of the included mini-HTAs

Study	Country of first author	Presence of HTA Professionals	Purpose of assessment	Type of disease	Assessment setting	Type of technology	Classification of journal	Funding
Ren 2021 (25)	China	Yes	Comparison of existing technologies	Extension code	General hospital	Drug	PKU+CSCD	Not reported*
Xue 2020 (30)	China	Yes	Comparison of existing technologies	Not reported	General hospital	Drug	PKU	Not reported
Fang 2022 (20)	China	No	Comparison of existing technologies	Diseases of the circulatory system	General hospital	Drug	Not applicable	Nonprofit
Duan 2021 (19)	China	No	Comparison of existing technologies	Mental, behavioural or neurodevelopmental disorders	General hospital	Drug	PKU	Nonprofit
Cao 2022 (16)	China	No	Comparsion of new with existing technologies	Neoplasms	Specialized hospital	Medical equipment	Not applicable	Not reported
Wang 2023 (28)	China	No	Comparison of existing technologies	Endocrine, nutritional or metabolic diseases	General hospital	Drug	Not applicable	Nonprofit
Cui 2020 (17)	China	No	Comparison of existing technologies	Not reported	General hospital	Drug	PKU+CSCD	Not reported
Li 2020 (24)	China	Yes	Comparison of existing technologies	Endocrine, nutritional or metabolic diseases	General hospital	Drug	PKU	Not reported
Ren 2023 (26)	China	No	Comparison of existing technologies	Not reported	General hospital	Drug	Not applicable	Nonprofit
Zhang 2022 (31)	China	Yes	Comparison of existing technologies	Diseases of the musculoskeletal system or connective tissue	General hospital	Drug	Not applicable	Nonprofit
Wang 2021 (27)	China	Not reported	Comparison of existing technologies	Endocrine, nutritional or metabolic diseases	General hospital	Drug	Not applicable	Nonprofit
Gu 2020 (10)	China	Not reported	Comparsion of new with existing technologies	Not reported	General hospital	Medical equipment	Not applicable	Nonprofit
Zhao 2018 (9)	China	No	Comparsion of new with existing technologies	Not reported	Specialized hospital	Medical equipment	Not applicable	Not reported
Wu 2021 (29)	China	No	Comparison of existing technologies	Diseases of the circulatory system	General hospital	Drug	Not applicable	Nonprofit
Huang 2023 (22)	China	No	Comparsion of new with existing technologies	Not reported	Specialized hospital	Drug	PKU	Not reported
Ju 2023 (23)	China	No	Comparison of existing technologies	Diseases of the nervous system	Specialized hospital	Drug	Not applicable	Not reported
Qiu 2019 (11)	China	No	Comparsion of new with existing technologies	Not reported	General hospital	Medical equipment	Not applicable	Not reported
Felipe 2016 (18)	Canada	Yes	Comparsion of new with existing technologies	Not reported	General hospital	Medical equipment	Not applicable	Nonprofit
Govender 2011 (21)	South Africa	Yes	Comparison of existing technologies	Not reported	General hospital	medical equipment	Q2	Not reported
Yang 2021 (4)	China	Not reported	Comparison of existing technologies	Not reported	General hospital	Medical equipment	Q3	Nonprofit
Martelli 2016 (6)	France	Yes	Comparison of existing technologies	Not reported	General hospital	Drug	Q1	Not reported

*Note:* HTA, Health Technology Assessment; PKU: A guide to the core journals of China of Perking Univeristy; CSCD: Chinese Science Citation Database; Q: Quartile

* refers to symptoms reported in the mini-HTA for which the ICD-11 classification does not provide a specific disease category.

### Mapping of included mini-HTA

**Figure 2.**
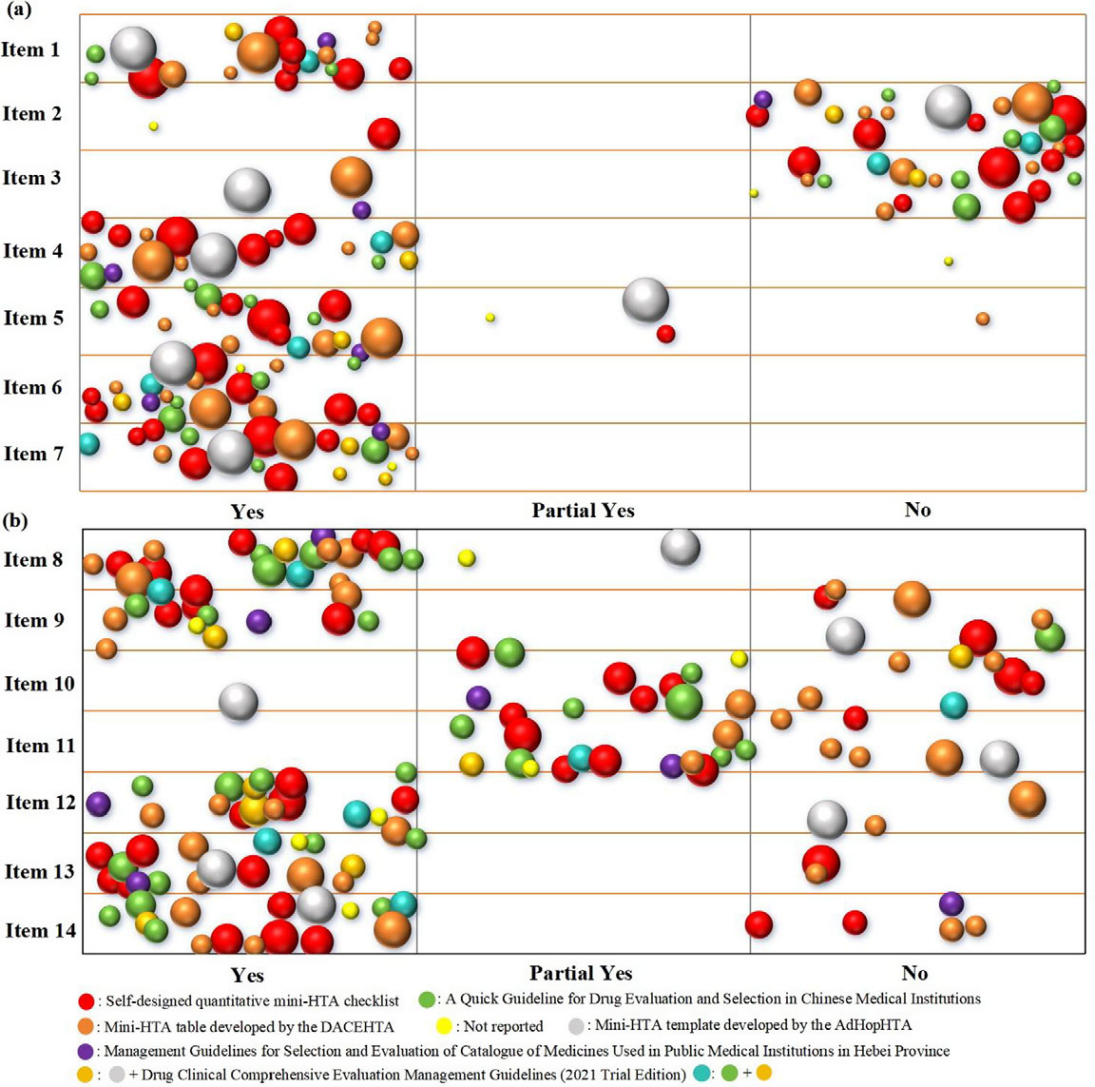
Bubble chart of included Mini-HTAs.

Based on the INAHTA checklist, as for item 1, all Mini-HTAs reported appropriate contact details (Figure 2). However, the reporting completeness was highly flawed in item 2, item 3, and item 11. Specially, more than 50.6 percent Mini-HTAs did not reported participant roles, conflict of interest statement, data sources, literature search strategies, and methods for assessing and analyzing data. The primary assessment criteria of included Mini-HTAs were self-designed quantitative Mini-HTA checklist and Mini-HTA table developed by DACEHTA (1), with 6 Mini-HTAs each, accounting for 28.6 percent. The number of authors contributing to Mini-HTAs ranged from 2 to 10, with the highest number of Mini-HTAs (n=8, 38.1 percent) contributed by 5 authors.

## Discussion

This evidence mapping provides a comprehensive review of current landscape of the 21 included Mini-HTAs, highlighting their characteristics, limitations, and areas for improvement. These Mini-HTAs covered seven disease categories, with endocrine, nutritional, and metabolic diseases being the most common. More than half of the Mini-HTAs did not report funding information, suggesting that they may have been conducted as part of routine decision-making processes within specific medical institutions without dedicated financial support. However, in our study, we found that only 33.3percent of Mini-HTAs involved HTA professionals, meaning that 66.7percent of Mini-HTAs were not written by professionals who specialize in HTA research and evaluation. This phenomenon may be related to the interdisciplinary nature of the HTA field, but it also suggests that we need to further strengthen the training and participation of HTA professionals to improve the overall quality of Mini-HTAs. Most Mini-HTAs emphasized comparisons of technologies and drug applications, with general hospitals serving as the primary implementation sites, which may indicate their limited integration into decision-making within other settings. Additionally, fewer than half were published in core Chinese journals or journals indexed in the JCR ranking system, limiting their academic visibility and broader impact. These findings suggest that while Mini-HTA activities have increased, their scientific rigor is often compromised due to the lack of HTA professionals, limited scope for assessment, and few publication in high-impact journals. Additionally, their integration into health system decision making beyond individual hospitals remains limited, as they are primarily conducted within individual hospitals rather than being widely adopted at a policy level. These challenges highlight the need to improve their scientific quality, expand their adoption in diverse healthcare settings, and enhance their overall impact.

The different roles of HTA across countries may contribute to variations in the publication and dissemination of HTA reports. A scoping review by Wu et al. (32) demonstrated that the impact of HTA on health decision-making varies across countries, influenced by different stakeholders, application methods, and objectives. In countries with publicly funded healthcare systems, such as Australia, the UK, and Canada, HTA primarily supports government decision-making and clinical practice. In contrast, in highly market-driven economies such as the USA, HTA is often conducted for commercial insurers, with relatively limited direct influence on public policy (32). Consequently, in Western countries, HTA reports are commonly published on government/medical institution websites or used internally by commercial entities, which may restrict their public dissemination.

In China, the application of HTA has been gradually shifting from purely academic research to evidence-based practice (33). This shift is accompanied by an increasingly widespread use of Mini-HTAs, which, compared to other countries, can be attributed to several factors. One key factor is that Mini-HTAs are designed based on the principles of evidence-based medicine, making them well-suited to the needs of HTA professionals. In addition, government policies promoting the integration of HTA into various decision-making scenarios have also played a significant role in driving its adoption. Our study further supports this trend, as we found that the first authors of the included Mini-HTAs were predominantly from China, a finding consistent with the studies of Yan et al. (34) and Li et al. (35), further supporting the notion that Mini-HTA has gained significant traction in the country.

According to assessment results of INAHTA checklist, four items were identified highly flawed. Firstly, specific responsibilities of individuals involved in the included Mini-HTAs (item 2) were not clearly defined. Mini-HTA should explicitly list all participants and their specific roles, including authors, committee member (if applicable), technical staff, administrative support personnel, etc. The absence of reporting item 2 can lead to an opaque assessment process, undermine the credibility of the research finding, and create accountability, which affects the ability of the stakeholders to effectively address issues in the decision-making proccess (36). Secondly, the included Mini-HTAs did not provide a conflict of interest statement (item 3). Conflicts of interest can impact the objectivity and validity of research findings, especially in studies founded by companies, where results may be biased toward the funders, affecting the scientific basis for decision-making and the reliability of clinical practice (15). Additionally, the included Mini-HTAs did not adequately describe the data sources and literature search strategies. Authors of Mini-HTAs should report all databases, websites, and other data sources, and provide a complete search strategy to ensure the reproducibility and verifiability of the research. This not only facilitates the update of Mini-HTAs but also helps improve the credibility of the research findings (37). Finally, the included Mini-HTAs did not provide the methods for assessing and analyzing data, a finding consistent with the conclusion of Kidholm et al (8). Authors of Mini-HTAs should describe these details to allow readers to replicate the research process and assess the appropriateness of the methods (38).

Notably, the overall assessment of completeness of reporting basic information of the included Mini-HTAs is generally positive. However, this could be influenced by the broad phrasing and somewhat vague criteria of the INAHTA checklist, which may make it challenging for relevant researchers to fully capture the specific assessment details. As a result, this might not always reflect the ture completeness of reported information (34). Since its publication in 2007, the INAHTA checklist has not been updated, nor has any other publicly available reporting guideline been introduced, which has led to limitations in its terminology and content in meeting current assessment needs (37). Additionlly, while the INAHTA checklist generally fulfills the basic reporting characteristics of HTA, its lack of features closely tied to mini-HTA may somewhat limit its practical application in Mini-HTA.

### Strengths and limitation

This evidence mapping has several strengths. Firstly, a comprehensive search was conducted across eight major Chinese and English-language databases, as well as major HTA-related websites, ensuring extensive coverage of available Mini-HTAs. Secondly, the literature selection, data extraction, and quality assessment were independently conducted by two researchers with expertise in evidence-based medicine, with any disagreements resolved through consensus or by involving a third researcher, ensuring the scientific rigor, thoroughness, and reliability of the study process. Thirdly, a bubble chart was used to visually present existing evidence from multiple critical dimensions, systematically illustrating key differences in reporting extent, number of authors involved, and assessment criteria, thereby identifying gaps in evidence and providing a valuable reference for future research in this area.

Nevertheless, there are several limitations to be acknowledged. Firstly, the findings are based solely on publications available before February 2024, and as new research emerges, the current results will need to be updated. Secondly, the inclusion of only Chinese and English language Mini-HTA reports, while representative, may somewhat limit the broader applicability and generalizability of the results.

## Conclusion

The number of publicly published Mini-HTAs has seen rapid growth in China, encompassing a wide range of diseases. However, their utilization remains limited in specialized hospitals and surgical settings. Furthermore, Mini-HTA reports often exhibit several deficiencies, including participant roles, conflict of interest statements, data sources, literature search strategies, and methods for data assessment and analysis. While the INAHTA checklist is widely used in HTA reporting, its application within the context of Mini-HTA may present certain limitations. Building on the findings of this study, a Mini-HTA reporting checklist will be developed in the future, with the aim of enhancing the standardization and rigor of Mini-HTAs, thereby maximizing their value and applicability.

## Supporting information

10.1017/S0266462325100585.sm001 Wang et al. supplementary material Wang et al. supplementary material

## Supplementary material

The supplementary material for this article can be found at http://doi.org/10.1017/S0266462325100585.
