# Supplementary material for: Trend, application, and reporting of Mini-health technology assessment: an evidence mapping
Source: Int J Technol Assess Health Care. 2025 Dec 4;42(1):e4. doi: 10.1017/S0266462325100585 (PMC12826859; doi:10.1017/S0266462325100585)
Supplement: Wang et al. supplementary material [file S0266462325100585sup001.docx]

**The list of major HTA-related website**

(1) National Institute for Health Research (NHS). Accessed from: <https://www.crd.york.ac.uk/CRDWeb>

(2) National Institute for Health and Care Excellence (NICE). Accessed from: https:/www.nice.org.uk/

(3) HAUTE AUTORITÉ DE SANTÉ (HAS). Accessed from: <https://www.has-sante.fr/portail/jcms/c_39085/en/recherche?portlet=c_39085&text=cusa&opSearch=&lang=en&portal=c_2566858>

(4) Pharmaceuticals and Medical Devices Agency (PMDA). Accessed from: <https://www.pmda.go.jp/english/>

(5) Ministry of Health and Welfare (MOHW). Accessed from: <https://www.mohw.go.kr/eng/index.jsp>

(6) Pharmaceutical Benefits Scheme (PBS). Accessed from: <https://www.pbs.gov.au/pbs/home>

(7) Canada's Drug AgencyL'Agence des médicaments du Canada (CDA-AMC). Accessed from: <https://www.cadth.ca/jakavi-myelofibrosis>

(8) Scottish Medicines Consortium (SMC). Accessed from: https:/www.scottishmedicines.org.uk/

(9) Servicos de Saúde do Governo da Regiäo Administrativa Especial de Macau. Accessed from: <https://ssm.gov.mo/portal/>

(10) Taiwan Center For Drug Evaluation. Accessed from: <https://www.cde.org.tw/>

**Search strategy:**

1. **CNKI** (https://www.cnki.net/)

TKA = ('卫生技术评估' + '卫生技术评价' + 'HTA') * ('迷你' + '微型' + 'Mini' + '医院')

1. **Wanfang Data** (https://www.wanfangdata.com.cn/index.html)

题名或关键词:("卫生技术评估" OR "卫生技术评价" OR "HTA") AND 题名或关键词:("迷你" OR "微型" OR "Mini" OR "医院")

1. **VIP** (https://www.cqvip.com/)

M = ("卫生技术评估" OR "卫生技术评价" OR "HTA") AND M = ("迷你" OR "微型" OR "Mini" OR "医院")

1. **CBM** (https://www.sinomed.ac.cn/zh/index.jsp?type=wx)

#1 "卫生技术评估"[常用字段:智能] OR "卫生技术评价"[常用字段:智能] OR "HTA"[常用字段:智能]

#2 "迷你"[常用字段:智能] OR "微型"[常用字段:智能] OR "Mini"[常用字段:智能] OR "医院"[常用字段:智能]

#3 #1 AND #2

**(5) PubMed** (https://pubmed.ncbi.nlm.nih.gov/)

#1 "Technology Assessment, Biomedical"[Mesh] OR "Biomedical Technology Assessment"[Title/Abstract] OR "Biomedical Technology Assessments"[Title/Abstract] OR "Health Technology Assessment"[Title/Abstract] OR "Health Technology Assessments"[Title/Abstract] OR "HTA"[Title/Abstract])

#2 Mini[Title/Abstract] OR hospital*[Title/Abstract]

#3 #1 AND #2

**(6) Embase** (https://www.embase.com/landing?status=grey)

#1 'biomedical technology assessment'/exp OR 'Biomedical Technology Assessment':ti,ab OR 'Biomedical Technology Assessments':ti,ab OR 'Health Technology Assessment':ti,ab OR 'Health Technology Assessments':ti,ab OR 'HTA':ti,ab

#2 Mini:ti,ab OR hospital*:ti,ab

#3 #1 AND #2

1. **Web of Science** (https://www.webofscience.com/wos/author/author-search)

#1 TS = ("Biomedical Technology Assessment" OR "Biomedical Technology Assessments" OR "Health Technology Assessment" OR "Health Technology Assessments" OR "HTA")

#2 TS = (Mini OR hospital*)

#3 #1 AND #2

1. HTA database (https://database.inahta.org/)

#1 "Biomedical Technology"[mh] OR "Biomedical Technology Assessment"[abs] OR "Biomedical Technology Assessments"[abs] OR "Health Technology Assessment"[abs] OR "Health Technology Assessments"[abs] OR "HTA"[abs]

#2 Mini[abs] OR hospital*[abs]

#3 #1 AND #2
